# Quantitative structure–activity relationship study of amide derivatives as xanthine oxidase inhibitors using machine learning

**DOI:** 10.3389/fphar.2023.1227536

**Published:** 2023-06-29

**Authors:** Xiaoda Yang, Hongshun Qiu, Yuxiang Zhang, Peijian Zhang

**Affiliations:** College of Computer Science and Technology, Qingdao University, Qingdao, China

**Keywords:** xanthine oxidase inhibitor, quantitative structure activity relationship, amide derivatives, XGBoost, support vector regression, random forest, particle swarm optimization

## Abstract

The target of the study is to predict the inhibitory effect of amide derivatives on xanthine oxidase (XO) by building several models, which are based on the theory of the quantitative structure–activity relationship (QSAR). The heuristic method (HM) was used to linearly select descriptors and build a linear model. XGBoost was used to non-linearly select descriptors, and radial basis kernel function support vector regression (RBF SVR), polynomial kernel function SVR (poly SVR), linear kernel function SVR (linear SVR), mix-kernel function SVR (MIX SVR), and random forest (RF) were adopted to establish non-linear models, in which the MIX-SVR method gives the best result. The kernel function of MIX SVR has strong abilities of learning and generalization of established models simultaneously, which is because it is a combination of the linear kernel function, the radial basis kernel function, and the polynomial kernel function. In order to test the robustness of the models, leave-one-out cross validation (LOOCV) was adopted. In a training set, $R^{2}$ = 0.97 and RMSE = 0.01; in a test set, $R^{2}$ = 0.95, RMSE = 0.01, and $R_{cv}^{2}$ = 0.96. This result is in line with the experimental expectations, which indicate that the MIX-SVR modeling approach has good applications in the study of amide derivatives.

## 1 Introduction

Hyperuricemia is a chronic metabolic disorder caused by impaired purine metabolism (Yang et al., 2022; Zeng et al., 2022; Johnson et al., 2023). Excess serum uric acid induces the formation of monosodium uric acid crystal deposits, which eventually leads to gout. In addition, hyperuricemia is also associated with many other chronic diseases, such as cardiovascular diseases, hypertension, and kidney disease (Johnson et al., 2023).

Xanthine oxidase (XO) is a key rate-limiting enzyme in the purine metabolism pathway. It catalyzes the oxidation of hypoxanthine and xanthine to uric acid with reactive oxidants being released in the process (Cicero et al., 2021; Yang et al., 2022). Excessive oxidants may lead to an oxidative stress reaction in the cells, which can lead to cell damage, and excessive oxidants are involved in many pathological processes, such as diabetes, chronic heart failure, and atherosclerosis (Jin et al., 2022). Therefore, XO is an important therapeutic target not only for the treatment of hyperuricemia and gout but also for many diseases related to oxidative stress.

Current therapeutic approaches for gout are mainly based on lowering serum uric acid levels; these approaches include the inhibition of XO, promotion of uric acid excretion, or alkalinization of urine (Fathallah-Shaykh and Cramer, 2014; Feng et al., 2022). One effective treatment for patients with hyperuricemia is the use of XO inhibitors, which directly block the oxidation of hypoxanthine and xanthine to produce uric acid. Several XO inhibitors have been developed, such as clinically approved allopurinol and febuxostat (Kojima et al., 2016; Packer, 2020). However, side effects of these drugs have been observed during clinical applications, so it is important to find new XO inhibitors.

Amide derivatives are a newly discovered type of XO inhibitors that have significant research value. Although assessing the inhibitory effect of XO ( $IC_{50}$ ) is a time-consuming and labor-intensive process, models based on the quantitative structure–activity relationship (QSAR) theory can predict the biological activity of new compounds precisely and quickly by constructing the quantitative relationship between chemical structure and biological activity (Si et al., 2021a; Chen et al., 2021). By constructing quantitative relationships and machine learning techniques, researchers can explore large datasets and accurately and quickly predict the biological activity of new compounds, which is of great significance for developing new drugs and saving human and material resources (Chen et al., 2022).

In this study, QSAR models were established by linear and non-linear methods based on descriptors selected by the heuristic method (HM) and XGBoost methods. Comparing the performance of models developed by linear regression, support vector regression (SVR), and random forest (RF) regression, it was observed that the model built by mix kernel SVR exhibits the best predictive ability and robustness.

## 2 Materials and methods

### 2.1 Data preparation

The compounds listed in Table 1 were taken from the following papers: Tu et al., 2021; Zhang et al., 2021; Zhang et al., 2022. All $IC_{50}$ values were measured in the same experimental environment. The compounds were randomly divided into training and test sets, which contain 44 compounds and 10 compounds, respectively.

**TABLE 1:** *In vitro* XO inhibitory potency of compounds.

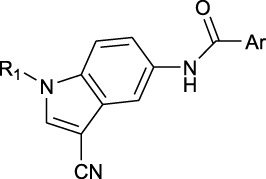						
Compound	$R_{1}$ group	Ar		$IC_{50}$ (μM)	-lg ( $IC_{50}$ )	MIX SVR
1	Propyl	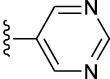		16.17	−1.21	−1.12
2	Propyl	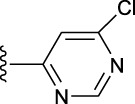		0.13	0.89	0.89
3	Propyl	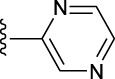		0.06	0.89	0.89
4	Propyl	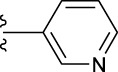		6.45	−0.81	−0.81
5	Propyl	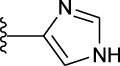		0.018	1.74	1.76
6	Propyl	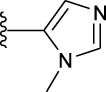		5.12	−0.71	−0.71
7	Propyl	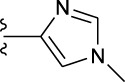		2.05	−0.31	−0.31
8*	Propyl	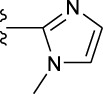		1.06	−0.03	−0.02
9	Propyl	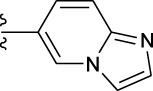		7.36	−0.87	−0.90
10*	Propyl	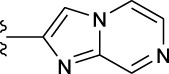		0.80	0.10	0.10
11	Propyl	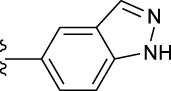		0.94	0.03	−0.15
12	Benzyl	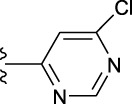		3.11	−0.49	−0.49
13	Benzyl	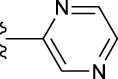		0.071	1.15	1.14
14	Benzyl	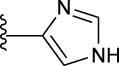		0.022	1.66	1.54
15	Benzyl	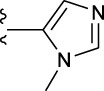		12.10	−1.08	−0.61
16*	Benzyl	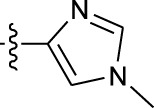		3.33	−0.52	−0.50
17	Benzyl	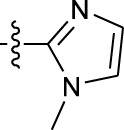		10.67	−1.03	−0.52
18	Benzyl	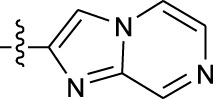		24.39	−1.39	−1.39
19	Benzyl	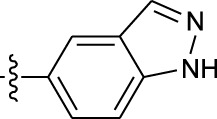		8.85	−0.95	−0.82
20	Cyclopentyl	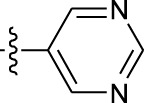		7.26	−0.86	−0.86
21	Cyclopentyl	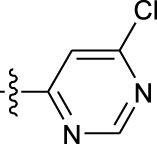		0.25	0.60	0.60
22*	Cyclopentyl	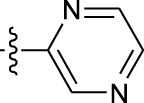		0.073	1.14	1.03
23	Cyclopentyl	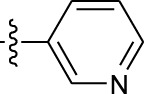		1.58	−0.20	−0.15
24*	Cyclopentyl	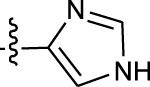		0.026	1.59	1.46
25	Cyclopentyl	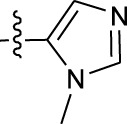		4.08	−0.61	−0.52
26	Cyclopentyl	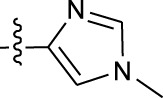		0.55	0.26	−0.11
27	Cyclopentyl	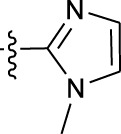		1.71	−0.23	−0.23
28*	Cyclopentyl	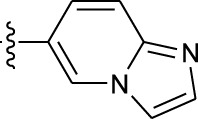		1.68	−0.52	−0.50
29	Cyclopentyl	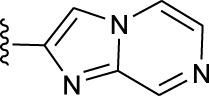		0.31	0.51	0.15
30	Cyclopentyl	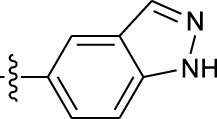		3.06	−0.49	−0.49

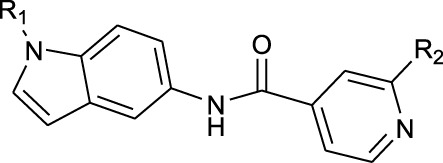						
Compound	$R_{1}$ group	$R_{2}$ group	$R_{3}$ group	$IC_{50}$ (μM)	-lg ( $IC_{50}$ )	MIX SVR
31	H	H	CN	8.59	−0.93	−0.89
32	Ethyl	H	CN	7.89	−0.90	−0.90
33	Propyl	H	CN	7.34	−0.88	−0.88
34	Isopropyl	H	CN	4.43	−0.65	−0.65
35	Allyl	H	CN	7.65	−0.88	−0.88
36	Prop-2-yn-1-yl	H	CN	1.18	−0.07	−0.10
37	Benzyl	H	CN	12.46	−1.10	−0.94
38	Cyclopentyl	H	CN	0.73	0.14	−0.15
39*	H	Cl	CN	4.30	−0.63	−0.63
40*	Propyl	Cl	CN	16.19	−1.21	−1.18
41	H	F	CN	6.95	−0.84	−0.63
42*	Propyl	F	CN	12.64	−1.10	−1.09
43	Allyl	F	CN	21.79	−1.34	−1.33
44	Prop-2-yn-1-yl	F	CN	5.04	−0.70	−0.54
45	Cyclopentyl	F	CN	12.64	−1.10	−0.86

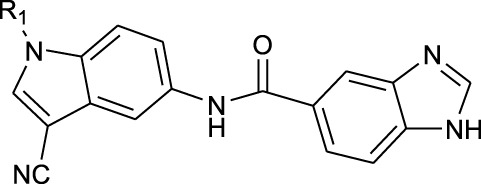						
Compound	$R_{1}$ group			IC_50_ (μM)	-lg (IC_50_)	MIX SVR
46	H			3.52	−0.55	−0.55
47	Methyl			4.49	−0.65	−0.66
48	Ethyl			2.16	−0.33	−0.33
49	Propyl			2.17	−0.34	−0.34
50	Isopropyl			4.25	−0.63	−0.53
51*	Allyl			2.04	−0.31	−0.31
52	Prop-2-yn-1-yl			4.27	−0.63	−0.52
53	Benzyl			1.52	−0.18	−0.19
54	Cyclopentyl			0.62	0.21	0.27

“*”: the compounds of the test set.

### 2.2 Descriptor calculation

The process of descriptor calculation is the basis of constructing QSAR models. First, the structures of these compounds were drawn using ChemDraw software. The result file was then imported into HyperChem and optimized using MM+ in the molecular mechanics force field. Second, the semi-empirical method was used to carry out further optimization so that the compound could have the lowest energy. According to the principle of minimum potential energy, molecular stability is enhanced by minimizing the energy state of a system, with lower energy states resulting in greater stability. After this process, the corresponding result file was sent to MOPAC software to change the format. Finally, five classes of descriptors were calculated using CODESSA software. These classes include topological, constitutional, geometrical, quantum chemical, and electrostatic descriptors (Katritzky et al., 2006).

### 2.3 Linear model by the heuristic method

The heuristic method, an efficient approach for descriptor selection and linear model construction, has no limitation to the size of the dataset (He et al., 2014; Si et al., 2021b). Before executing the model establishment algorithm, descriptors should be pre-selected under the guidance of the following principles: non-generic descriptors and descriptors with a constant value should be removed, and descriptors with a large correlation coefficient (greater than 0.8) should also be discarded (Si et al., 2021b).

The descriptor selection process by the HM involves calculating intercorrelations between all descriptors and identifying pairs with high regression coefficients but low correlation coefficients. Non-collinear descriptors are added to these pairs to perform higher-order regression treatments, and the final equation is selected based on the maximum Fisher criterion and the highest cross-validated correlation coefficient used in the linear model (Katritzky et al., 1995).

### 2.4 Non-linearly selecting descriptors by XGBoost

In order to improve the performance and robustness of the non-linear model, a non-linear method is used to select descriptors. XGBoost can be adopted to perform non-linear dimensionality reduction, which is mainly based on feature importance evaluation (Chen et al., 2020). There are two ways to calculate feature importance in XGBoost: based on the coverage or on the split gain.

The coverage method determines feature importance by calculating the sum of the number of times that each feature occurs in all tree nodes. The higher the coverage is, the more important the feature is considered to be. This method is useful when working with datasets that have many features as it provides a quick way of identifying the most relevant features.

The split gain method determines feature importance by calculating the information gain of each feature when the decision tree is split. The higher the split gain, the more important the feature is considered to be. This method is especially useful when working with datasets that have complex relationships between the features as it can capture subtler interactions than the coverage method.

The split gain method is the default method used by XGBoost if no extra settings are added because the split gain method is generally more effective at capturing the underlying relationships between the features in the dataset. The coverage method provides a quick and simple way to evaluate feature importance, but it tends to favor features with a high cardinality. However, both methods have their own strengths and weaknesses, and the choice of which method to use will depend on the specific dataset and the goals of the analysis.

### 2.5 Non-linear model by the random forest

Random forest is a popular bagging algorithm, which combines multiple decision trees to make accurate predictions in a regression. This algorithm creates multiple decision trees by randomly selecting subsets of features and data samples, and then averaging their outputs to obtain the final prediction. In this process, as the depth of the tree increases, the entropy of sample types under decision tree branches will also increase, which will seriously affect the efficiency of the decision tree and increase the risk of overfitting. Therefore, the random forest reduces the overfitting probability and improves the generalization ability of the model by pruning and other methods. These methods remove unnecessary nodes or branches from the decision tree.

Random forest has the advantages of fast training and good performance in a classification, and it can handle non-linear data very well. However, compared to other regression methods like SVR, the performance of random forests may be affected by outliers in the data, which can result in reduced robustness. Therefore, its performance somewhat depends on the dataset.

### 2.6 Non-linear model by SVR

The support vector machine (SVM) (Vapnik, 1995) is a classification algorithm proposed by Vapnik et al., in 1990. The SVM uses kernel tricks to map data into higher dimensions and find optimal hyperplanes, allowing it to handle non-linear problems with great accuracy. The SVM aims to minimize the difference between predicted and actual values and, at the same time, maximize the distance between the hyperplane and sample points. This approach was later extended to support vector regression for regression problems, which has demonstrated strong performance. Generalization performance and robustness of regression models based on the SVR approach can be enhanced (Tang et al., 2022; Helmy et al., 2023; Ying et al., 2023).

Because SVR is very sensitive to the scale of the input data, normalization must be executed before the SVR process to ensure the accuracy and robustness of the model to be established.

After performing normalization, the next step is to establish models by SVR. First, the original problem is transformed into a dyadic problem while satisfying the Karush–Kuhn–Tucker (KTT) principle, and the Lagrange multiplier method is used on the basis of the dyadic problem. Ultimately, the problem can be simplified to solve the following quadratic convex programming problems:

$$\min\;\frac{1}{2}\sum_{i,j=1}^{m}y^{\left(i\right)}y^{\left(j\right)}\alpha_{i}\alpha_{j}<x^{\left(i\right)},x^{\left(j\right)}>-\sum_{i=1}^{m}\alpha_{i},$$ (1)

$$s.t.\;\alpha_{i}\geq 0\left(\forall i\right),\sum_{i=1}^{m}\alpha_{i}y^{\left(i\right)}=0.$$ (2)

The initial SVR excelled in solving linear problems rather than non-linear problems, so the kernel method was then introduced to solve non-linear problems. It is an easy way that uses the kernel function to calculate the inner product in Eq. 1 after mapping it to a higher dimensional space. Common kernel functions include linear kernel functions, polynomial kernel functions, and radial basis kernel functions. We replace $<x^{\left(i\right)},x^{\left(j\right)}>$ by $\varphi {\left(x^{\left(i\right)}\right)}^{T}\varphi \left(x^{\left(j\right)}\right)=\kappa \left(x^{\left(i\right)},x^{\left(j\right)}\right)=\kappa_{ij}$ , where φ is the mapping function that maps vectors from a low-dimensional to a high-dimensional space. Then, the problem is expressed as follows:

$$\min\;\frac{1}{2}\sum_{i,j=1}^{m}y^{\left(i\right)}y^{\left(j\right)}\alpha_{i}\alpha_{j}\kappa_{i,j}-\sum_{i=1}^{m}\alpha_{i},$$ (3)

$$s.t.\;\alpha_{i}\geq 0\left(\forall i\right),\sum_{i=1}^{m}\alpha_{i}y^{\left(i\right)}=0.$$ (4)

Moreover, a soft-interval SVM was introduced to enhance the robustness of the SVM, which controls the tolerance of the SVM to noise by introducing relaxation variables and penalty factors.

### 2.7 PSO parameter optimization (Eberhart and Kennedy, 2023)

Since the RF and MIX-SVR algorithm has many parameters that are not independent, particle swarm optimization (PSO) was used for parameter optimization in RFs and MIX SVR.

PSO, an optimization algorithm based on population intelligence, was first proposed by Eberhart and Kennedy in 1995. PSO is a vector-based method, which achieves optimal search by sharing information among populations, and all iterations update the position vector and velocity vector, according to the following two equations:

$$v=w*v+C1*r1*\left(pbest-x\right)+C2*r2*\left(gbest-x\right),$$ (5)

x=x+v, (6)

where w is the inertia coefficient, pbest is the historical optimal position of each particle, and gbest is the position of the best advantage of the whole population.

Based on the traditional PSO algorithm, some improvements have been made. Because some parameters can only be integer values, while some parameters take values in the whole real number domain, mixed optimization is introduced, which means that the improved algorithm will automatically match, whether it is for integer optimization or real number optimization. The principle of solving the integer search problem is based on the original real number search problem, which involves rounding the real number x generated by each operation to the nearest integer to participate in the next operation.

In addition, the improvement of PSO also involved the method of linear decreasing weights, which means the pace is very large at first but decreases with each epoch. The weight varies according to Eq. 7, where $w_{init}$ is the initial weight, $w_{end}$ is the end weight, NGEN is the total number of iterations, and gen is the current iteration. When the inertia weight is large, it has a large exploration space; however, it is easy to miss the optimal solution. When the inertia weight is small, it is favorable for seeking local optimization. Therefore, a large weight is used at the beginning of all iterations for a wide range of search, and as the number of iterations increases, the weight decreases linearly, which is more favorable for the local optimization search.

$$w=\left(w_{init}-w_{end}\right)*\left(NGEN-gen\right)/NGEN+w_{end}.$$ (7)

## 3 Results

### 3.1 Linear model by the HM

**FIGURE 1:**
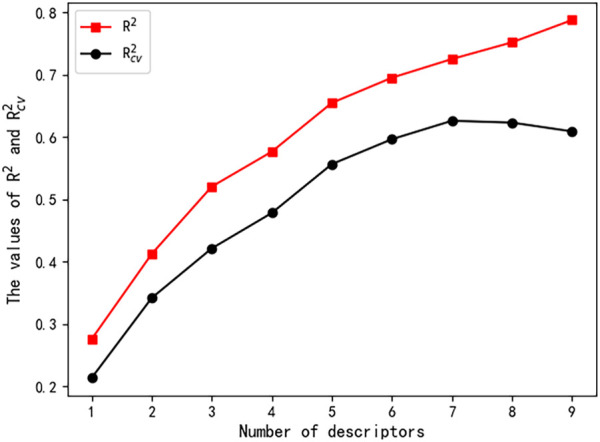
Influence of the number of descriptors on $R^{2}$ and $R_{cv}^{2}$ .

A total of 646 descriptors were calculated according to the descriptor calculation step described in the descriptor calculation subsection. The number of descriptors in linear models was increased from one to seven, and the corresponding $R^{2}$ and $R_{cv}^{2}$ values were recorded. As shown in Figure 1, the $R_{cv}^{2}$ value stopped increasing when descriptor numbers reached seven. The seven selected descriptors and their correlation coefficients are shown in Table 2 and Table 3, respectively.

**TABLE 2:** Physical–chemical meaning of the chosen descriptors.

Physical–chemical meaning	Abbreviation
FPSA-3 Fractional PPSA (PPSA-3/TMSA) (Zefirov’s PC)	FFP
Min total interaction for a C–H bond	MTI
Min exchange energy for a C–C bond	MEE
Relative number of F atoms	RNO
HA-dependent HDCA-2/TMSA (Zefirov’s PC)	HDH
HACA-1/TMSA (Zefirov’s PC)	HTZ
Count of H-acceptor sites (Zefirov’s PC)	CHZ

**TABLE 3:** Correlation matrix of descriptors by the HM.

Descriptor	FFP	MTI	MEE	RNO	HDH	HTZ	CHZ
FFP		0.32	0.13	0.09	0.66	0.02	0.54
MTI			0.00	0.04	0.58	0.35	0.04
MEE				−0.09	0.12	−0.00	0.02
RNO					0.13	0.17	−0.43
HDH						0.47	0.19
HTZ							−0.46
CHZ							

**FIGURE 2:**
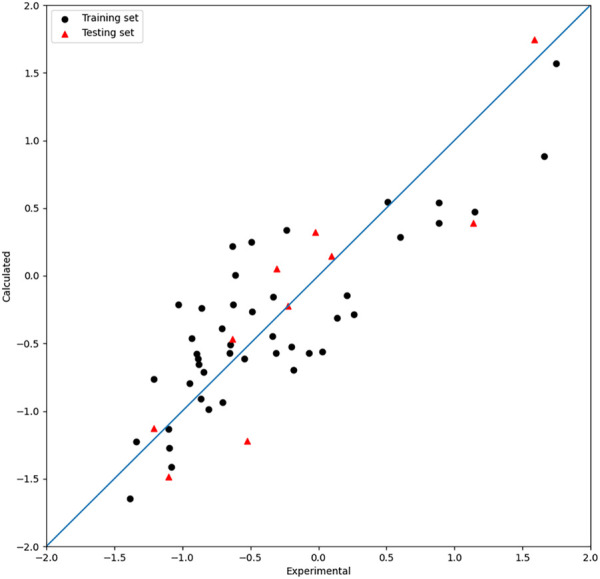
Plot of the experimental and calculated −lg ( ${\text{IC}}_{50}$ ) of the HM model.

Based on the seven descriptors selected by the HM, the linear model was built and shown in Eq. 8. As shown in Figure 2, the linear model did not achieve satisfactory results. Moreover, as shown in Figure 1, the $R^{2}$ value continues to increase with the increase in the descriptors’ number, but the $R_{cv}^{2}$ value reaches 0.6 and stops increasing, which indicates that the linear model is not robust.

The ${\text{IC}}_{50}$ value is influenced by various factors; most biochemistry data may be not linear (Wang et al., 2020), so non-linear models were attempted.

$$\begin{array}{l} {-\mathrm{lg}(\text{IC}}_{50})=325.03*\text{FFP}-3.16*\text{MTI}-4.69*\text{MEE}-46.34*\text{RNO}+3899.61*\text{HDH}\\ -168.41*\text{HTZ}-0.58*\text{CHZ}+57.74. \end{array}$$ (8)

### 3.2 Non-linearly selecting descriptors by XGBoost

Non-linear descriptor selection can better capture complex non-linear relationships in the data (Yamada et al., 2018). As selecting descriptors by the HM is a linear method, XGBoost is a non-linear method by contrast, so it was chosen to select a new group of descriptors before building a non-linear model.

**FIGURE 3:**
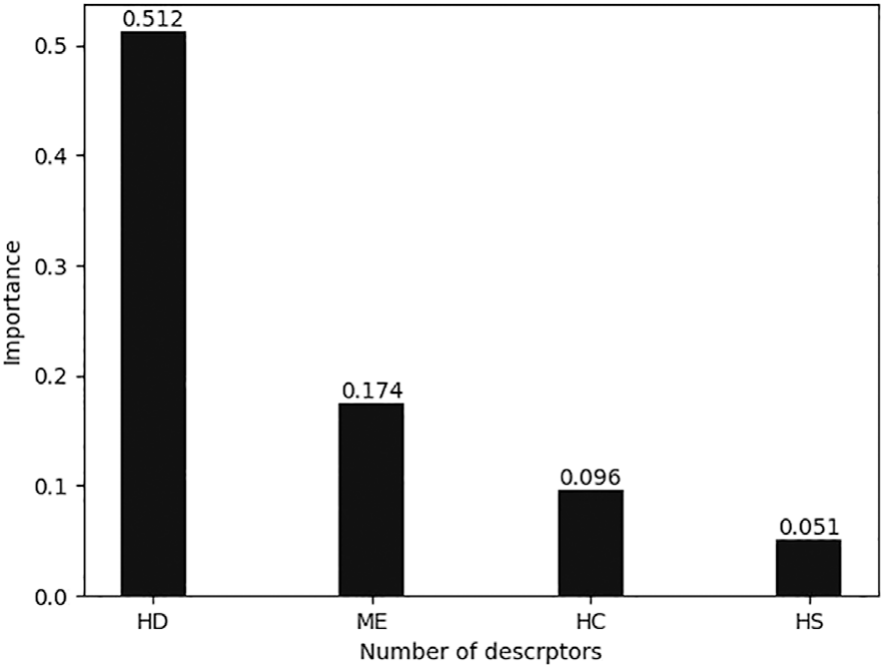
Importance of descriptors selected by XGBoost.

All calculated descriptors exported from CODESSA were pre-processed, and non-generic descriptors were removed. As mentioned previously, XGBoost selects descriptors by calculating the importance of each descriptor based on the split gain method. Figure 3 shows the importance of the highest four descriptors. The cumulative importance of the four descriptors reached 85%, which can already express the complete characteristics of the dataset. Adding the fifth descriptor will not significantly increase the expressiveness, but will increase the risk of overfitting; therefore, the four descriptors shown in Table 4 were chosen for the sake of balance.

**TABLE 4:** Physical–chemical meaning of the chosen descriptors.

Physical–chemical meaning	Number representation
HA-dependent HDCA-2/TMSA (Quantum-Chemical PC)	HD
Maximum electronic repulsion for a hydrogen atom	ME
HACA-2 (Quantum-Chemical PC)	HC
HASA-1 (Quantum-Chemical PC)	HS

The correlation matrix is shown in Table 5, so as to initially verify the validity of the selected descriptors. As shown in Table 5, the correlation coefficient of any two descriptors is less than 0.5, which means that a non-linear method can be built using these descriptors.

**TABLE 5:** Correlation matrix of descriptors by XGBoost.

Descriptor	HD	ME	HC	HS
HD		0.1	0.32	0.17
ME			−0.05	−0.19
HC				−0.29
HS				

### 3.3 Non-linear model by RFs

The RF method is a popular regression method, which performs well in many research fields (Gao et al., 2022). Four important parameters should be determined when building models using this method. Their names and functions are as follows:

(1) Number of trees (NT): Increasing the number can improve the model performance at the cost of computational complexity.
(2) Maximum depth of the tree (MD): The purpose of this parameter is to prevent overfitting by controlling the depth of the tree. Increasing the maximum depth can improve the model performance at the cost of the risk of overfitting.
(3) Minimum number of samples required to split internal nodes (MS): The purpose of this parameter is to control the minimum size of leaf nodes to avoid overfitting. Increasing this parameter may lead to underfitting.
(4) Minimum number of samples required for leaf nodes (ML): It is used in the pre-pruning of the decision tree.

**FIGURE 4:**
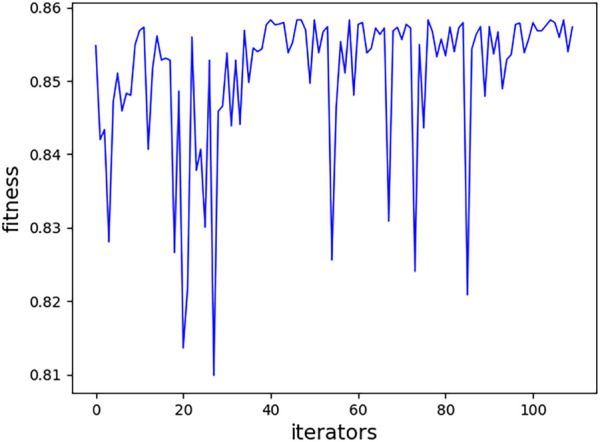
Fitness of each iteration.

**FIGURE 5:**
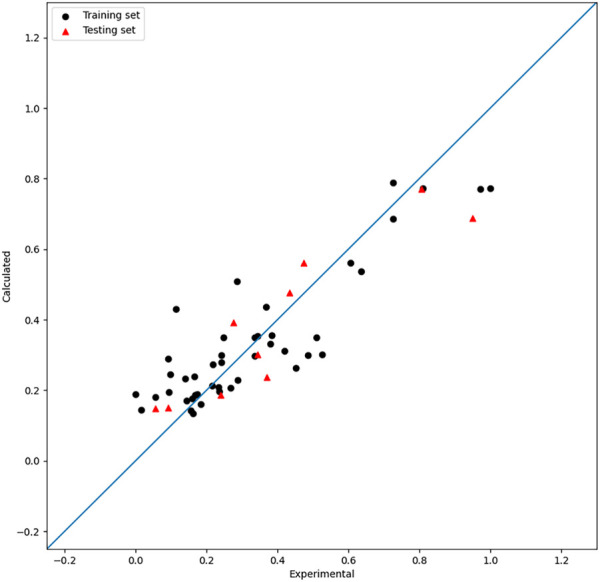
Plot of the experimental and calculated −lg ( ${\text{IC}}_{50}$ ) of the RF model.

The PSO method is adopted to tune the hyperparameters; one relatively good set of parameters are NT = 522, MD = 13, MS = 7, and ML = 4. The tuning process of the model based on RFs by PSO is shown in Figure 4. The $R^{2}$ value of the training and test sets are 0.86 and 0.86, respectively. The RMSE values of the training and test sets are 0.01 and 0.01, respectively. The $R_{cv}^{2}$ value of the model by RFs is 0.66. The result is shown in Figure 5. As mentioned previously, the PSO algorithm here adopts the idea of decreasing linear weights and performs hybrid parametric search optimization, which further improves its parametric searching efficiency.

### 3.4 Non-linear models by single-kernel SVR

**FIGURE 6:**
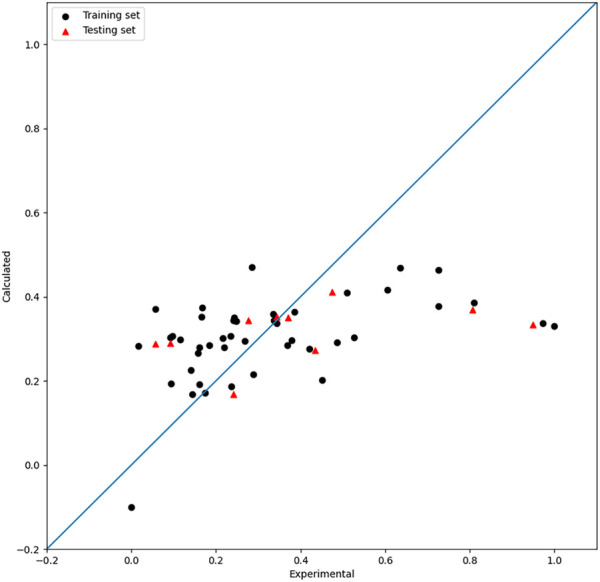
Plot of the experimental and calculated −lg ( ${\text{IC}}_{50}$ ) of the linear-SVR model.

**FIGURE 7:**
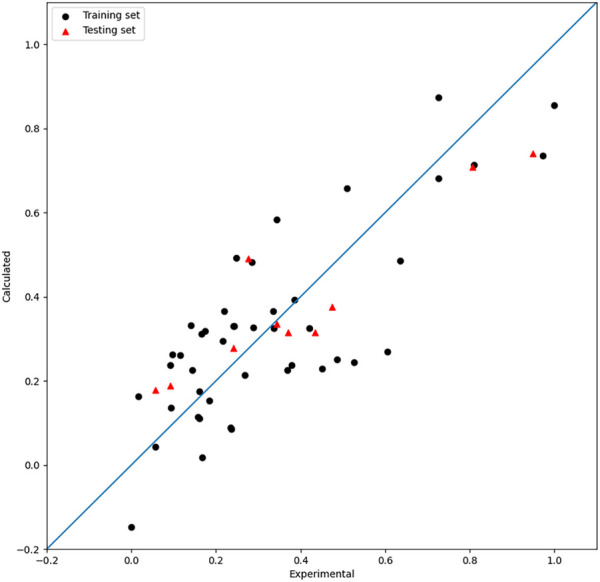
Plot of the experimental and calculated −lg ( ${\text{IC}}_{50}$ ) of the poly-SVR model.

**FIGURE 8:**
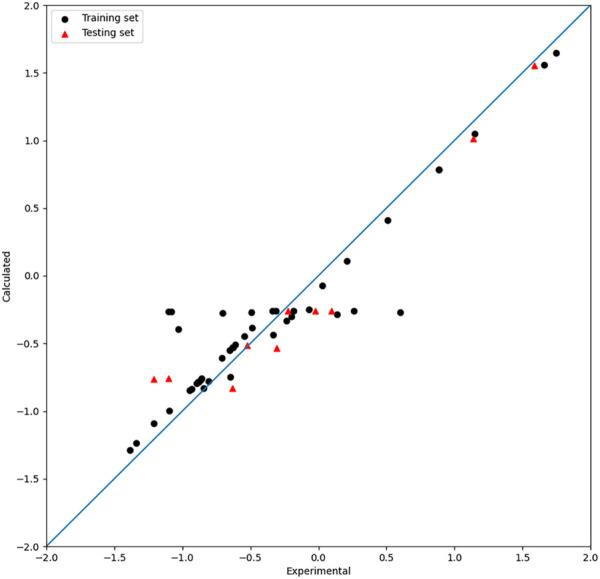
Plot of the experimental and calculated −lg ( ${\text{IC}}_{50}$ ) of the RBF-SVR model.

To ensure comparability between models, the same four descriptors selected by XGBoost were used in the models built by linear-kernel SVR, polynomial kernel function (poly)-kernel SVR, and radial basis kernel function (RBF)-kernel SVR. The $R^{2}$ values of the test set are 0.03, 0.79, and 0.91, for which the results are shown in Figure 6, Figure 7, and Figure 8, respectively. LOOCV was used to evaluate the model, and the $R_{cv}^{2}$ values of the models built by linear-kernel SVR, poly-kernel SVR, and RBF-kernel SVR are 0.53, 0.87, and 0.90, respectively.

As shown in the results, the three models by single-kernel functions did not perform well, which is related to their characteristics. So attempts were made to get better models by combining different kernels. The radial basis kernel function is strong in fitting, which increases the possibility of overfitting to some extent. The polynomial kernel function and the linear kernel function show good generalization performances, which increases the possibility of underfitting to some extent. MIX SVR is an attempt at combining the superiority of different kernels in order to build a model with both strong generalization and fitness abilities.

### 3.5 Non-linear model by mix-kernel SVR

For SVR, the choice of the kernel function has an obverse impact on the regression results. As mentioned previously in the single-kernel SVR part, the RBF kernel is good at fitting, while the linear and poly kernel are good at generalization. The kernel function designed in this paper is a proportional combination of these three kernel functions, which improves both the generalization performance and robustness of the model. The mix kernel function is expressed as follows in Eq. 9 (Shawe-Taylor and Cristianini, 2004):

$$\kappa =w\cdot \kappa_{rbf}+\left(1-w\right)\cdot \kappa_{poly}+b\cdot \kappa_{linear}.$$ (9)

**FIGURE 9:**
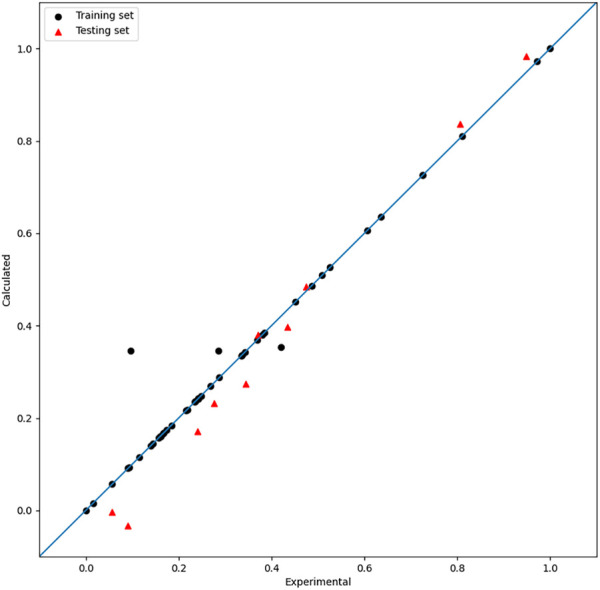
Plot of the experimental and calculated −lg ( ${\text{IC}}_{50}$ ) of the MIX-SVR model.

After adjusting the parameters using PSO, optimum C = 206.79, gamma = 50.47, p = 2, w = 0.04, and b = 0.03. The $R^{2}$ values of the training and test sets are 0.97 and 0.95, respectively, and the RMSE of the training set is 0.01, while it is 0.01 for the test set. Furthermore, the model has a robust cross-validation result of 0.96. By comparison, the mixed-kernel function performs better than the single-kernel function in building regression models. The contrast between the experimental and calculated results is shown in Figure 9. Inverse normalization is carried out, and the prediction value is shown in Table 1.

## 4 Discussion

A visual comparison is shown in Table 6. As shown in the table, the $R_{\text{cv}}^{2}$ value of the model based on mix-kernel SVR is the highest, which also illustrates the robustness of the model. Moreover, the result further shows that the XGBoost descriptor selection method is valid.

**TABLE 6:** Comparison of the results between different modeling methods.

Modeling method	$R_{cv}^{2}$	Training set		Test set	
		$R^{2}$	RMSE	$R^{2}$	RMSE
Linear model	0.60	0.69	0.17	0.79	0.14
MIX-SVR model	0.96	0.97	0.01	0.95	0.01
RBF-SVR model	0.90	0.85	0.08	0.91	0.06
Poly-SVR model	0.87	0.61	0.02	0.79	0.01
Linear-SVR model	0.53	0.20	0.04	0.03	0.07
RF model	0.66	0.86	0.01	0.86	0.01

The MIX-SVR method can effectively fit the data without overfitting because it takes advantage of the complementary features of polynomial and radial basis kernel functions. Overall, the mix-kernel SVR method is a promising approach for use in various applications requiring accurate and reliable regression analysis results.

The descriptor-labeled HD is the surface area of the hydrogen donor divided by the total area of the molecule. It has been determined to hold the greatest significance among the four descriptors being evaluated. This suggests that it is likely a variable that is strongly associated with the dependent variable or that it possesses a high degree of predictive power. In addition, this descriptor may also enhance model performance by collaborating with other descriptors. The second descriptor, ME, has the maximum electron–electron repulsive force between electron clouds in a hydrogen atom. The inter-electron repulsion affects the reaction rate to some extent. HS is the surface area of the hydrogen-bonded receptors in the molecule. Molecules with more hydrogen-bonded receptors can form tighter bindings to target proteins. HC is the surface charge of the hydrogen bonding donor atom. They are both quantum chemical descriptors, which can be used to predict their chemical properties.

## 5 Conclusion

The performance and robustness of the models constructed by mix-kernel SVR have been verified in predicting the ${\text{IC}}_{50}$ value of the related derivatives. This suggests that mix-kernel SVR could potentially serve as a valuable tool in reducing the cost and time required for amide derivative drug development. Additionally, this study has identified four key descriptors that appear to affect the activity of drugs used to treat gout and related diseases. These descriptors are HA-dependent HDCA-2/TMSA (Quantum-Chemical PC), the max e–e repulsion for a H atom, HACA-2 (Quantum-Chemical PC), and HASA-1 (Quantum-Chemical PC). By understanding the roles of these descriptors in the activity of these types of drugs, researchers may gain insights into the mechanisms of action and potential avenues for further drug design and development (Chen and Guestrin, 2023).

## Data Availability

The original contributions presented in the study are included in the article/Supplementary Material; further inquiries can be directed to the corresponding author.
