# Supplementary material for: Quantitative structure–activity relationship study of amide derivatives as xanthine oxidase inhibitors using machine learning
Source: Front Pharmacol. 2023 Jun 29;14:1227536. doi: 10.3389/fphar.2023.1227536 (PMC10339742; doi:10.3389/fphar.2023.1227536)

Supplementary Material

**Table 1. In vitro XO inhibitory potency of compounds**

|  | | | | | | |
| --- | --- | --- | --- | --- | --- | --- |
| Compounds | $R_{1}$ groups | Ar | | $IC_{50}$(μM) | -lg($IC_{50}$) | MIX-SVR |
| 1 | Propyl |  | | 16.17 | -1.21 | -1.12 |
| 2 | Propyl |  | | 0.13 | 0.89 | 0.89 |
| 3 | Propyl |  | | 0.06 | 0.89 | 0.89 |
| 4 | Propyl |  | | 6.45 | -0.81 | -0.81 |
| 5 | Propyl |  | | 0.018 | 1.74 | 1.76 |
| 6 | Propyl |  | | 5.12 | -0.71 | -0.71 |
| 7 | Propyl |  | | 2.05 | -0.31 | -0.31 |
| 8* | Propyl |  | | 1.06 | -0.03 | -0.02 |
| 9 | Propyl |  | | 7.36 | -0.87 | -0.90 |
| 10* | Propyl |  | | 0.80 | 0.10 | 0.10 |
| 11 | Propyl |  | | 0.94 | 0.03 | -0.15 |
| 12 | Benzyl |  | | 3.11 | -0.49 | -0.49 |
| 13 | Benzyl |  | | 0.071 | 1.15 | 1.14 |
| 14 | Benzyl |  | | 0.022 | 1.66 | 1.54 |
| 15 | Benzyl |  | | 12.10 | -1.08 | -0.61 |
| 16* | Benzyl |  | | 3.33 | -0.52 | -0.50 |
| 17 | Benzyl |  | | 10.67 | -1.03 | -0.52 |
| 18 | Benzyl |  | | 24.39 | -1.39 | -1.39 |
| 19 | Benzyl |  | | 8.85 | -0.95 | -0.82 |
| 20 | Cyclopentyl |  | | 7.26 | -0.86 | -0.86 |
| 21 | Cyclopentyl |  | | 0.25 | 0.60 | 0.60 |
| 22* | Cyclopentyl |  | | 0.073 | 1.14 | 1.03 |
| 23 | Cyclopentyl |  | | 1.58 | -0.20 | -0.15 |
| 24* | Cyclopentyl |  | | 0.026 | 1.59 | 1.46 |
| 25 | Cyclopentyl |  | | 4.08 | -0.61 | -0.52 |
| 26 | Cyclopentyl |  | | 0.55 | 0.26 | -0.11 |
| 27 | Cyclopentyl |  | | 1.71 | -0.23 | -0.23 |
| 28* | Cyclopentyl |  | | 1.68 | -0.52 | -0.50 |
| 29 | Cyclopentyl |  | | 0.31 | 0.51 | 0.15 |
| 30 | Cyclopentyl |  | | 3.06 | -0.49 | -0.49 |
|  | | | | | | |
| Compounds | $R_{1}$ groups | $R_{2}$ groups | $R_{3}$ groups | $IC_{50}$(μM) | -lg($IC_{50}$) | MIX-SVR |
| 31 | H | H | CN | 8.59 | -0.93 | -0.89 |
| 32 | Ethyl | H | CN | 7.89 | -0.90 | -0.90 |
| 33 | Propyl | H | CN | 7.34 | -0.88 | -0.88 |
| 34 | iso-Propyl | H | CN | 4.43 | -0.65 | -0.65 |
| 35 | Allyl | H | CN | 7.65 | -0.88 | -0.88 |
| 36 | Prop-2-yn-1-yl | H | CN | 1.18 | -0.07 | -0.10 |
| 37 | Benzyl | H | CN | 12.46 | -1.10 | -0.94 |
| 38 | Cyclopentyl | H | CN | 0.73 | 0.14 | -0.15 |
| 39* | H | Cl | CN | 4.30 | -0.63 | -0.63 |
| 40* | Propyl | Cl | CN | 16.19 | -1.21 | -1.18 |
| 41 | H | F | CN | 6.95 | -0.84 | -0.63 |
| 42* | Propyl | F | CN | 12.64 | -1.10 | -1.09 |
| 43 | Allyl | F | CN | 21.79 | -1.34 | -1.33 |
| 44 | Prop-2-yn-1-yl | F | CN | 5.04 | -0.70 | -0.54 |
| 45 | Cyclopentyl | F | CN | 12.64 | -1.10 | -0.86 |
|  | | | | | | |
| Compounds | $R_{1}$ groups | | | IC50(μM) | -lg(IC50) | MIX-SVR |
| 46 | H | | | 3.52 | -0.55 | -0.55 |
| 47 | Methyl | | | 4.49 | -0.65 | -0.66 |
| 48 | Ethyl | | | 2.16 | -0.33 | -0.33 |
| 49 | Propyl | | | 2.17 | -0.34 | -0.34 |
| 50 | iso-Propyl | | | 4.25 | -0.63 | -0.53 |
| 51* | Allyl | | | 2.04 | -0.31 | -0.31 |
| 52 | Prop-2-yn-1-yl | | | 4.27 | -0.63 | -0.52 |
| 53 | Benzyl | | | 1.52 | -0.18 | -0.19 |
| 54 | Cyclopentyl | | | 0.62 | 0.21 | 0.27 |

"*": The compounds of the test set

**Table 2. The physical-chemical meaning of the chosen descriptors**

| Physical-chemical meaning | Abbreviation |
| --- | --- |
| FPSA-3 Fractional PPSA ( PPSA-3/TMSA)[Zefirov's PC] | FFP |
| Min total interaction for a C-H bond | MTI |
| Min exchange energy for a C-C bond | MEE |
| Relative number of F atoms | RNO |
| HA dependent HDCA-2/TMSA[Zefirov's PC] | HDH |
| HACA-1/TMSA[Zefirov's PC] | HTZ |
| count of H-acceptor sites [Zefirov's PC] | CHZ |

**Table 3. Correlation matrix of descriptors by HM**

| Descriptor | FFP | MTI | MEE | RNO | HDH | HTZ | CHZ |
| --- | --- | --- | --- | --- | --- | --- | --- |
| FFP |  | 0.32 | 0.13 | 0.09 | 0.66 | 0.02 | 0.54 |
| MTI |  |  | 0.00 | 0.04 | 0.58 | 0.35 | 0.04 |
| MEE |  |  |  | -0.09 | 0.12 | -0.00 | 0.02 |
| RNO |  |  |  |  | 0.13 | 0.17 | -0.43 |
| HDH |  |  |  |  |  | 0.47 | 0.19 |
| HTZ |  |  |  |  |  |  | -0.46 |
| CHZ |  |  |  |  |  |  |  |

**Table 4. The physical-chemical meaning of the chosen descriptors**

| Physical-chemical meaning | Number representation |
| --- | --- |
| HA dependent HDCA-2/TMSA[Quantum-Chemical PC] | HD |
| Max e-erepulsion for a Hatom | ME |
| HACA-2[Quantum-Chemical PC] | HC |
| HASA-1[Quantum-Chemical PC] | HS |

**Table 5. Correlation matrix of descriptors by XGBoost**

| Descriptor | HD | ME | HC | HS |
| --- | --- | --- | --- | --- |
| HD |  | 0.1 | 0.32 | 0.17 |
| ME |  |  | -0.05 | -0.19 |
| HC |  |  |  | -0.29 |
| HS |  |  |  |  |

**Table 6. Comparision of results between different modeling method**

| Modeling method | $\text{R}_{cv}^{2}$ | Training set | | Test set | |
| --- | --- | --- | --- | --- | --- |
|  |  | $\text{R}^{2}$ | RMSE | $\text{R}^{2}$ | RMSE |
| Linear model | 0.60 | 0.69 | 0.17 | 0.79 | 0.14 |
| MIX-SVR model | 0.96 | 0.97 | 0.01 | 0.95 | 0.01 |
| RBF-SVR model | 0.90 | 0.85 | 0.08 | 0.91 | 0.06 |
| Poly-SVR model | 0.87 | 0.61 | 0.02 | 0.79 | 0.01 |
| Linear-SVR model | 0.53 | 0.20 | 0.04 | 0.03 | 0.07 |
| RF model | 0.66 | 0.86 | 0.01 | 0.86 | 0.01 |

**Figure 1.** **Influence of the number of descriptors on $\text{R}^{2}$and** $\text{R}_{cv}^{2}$


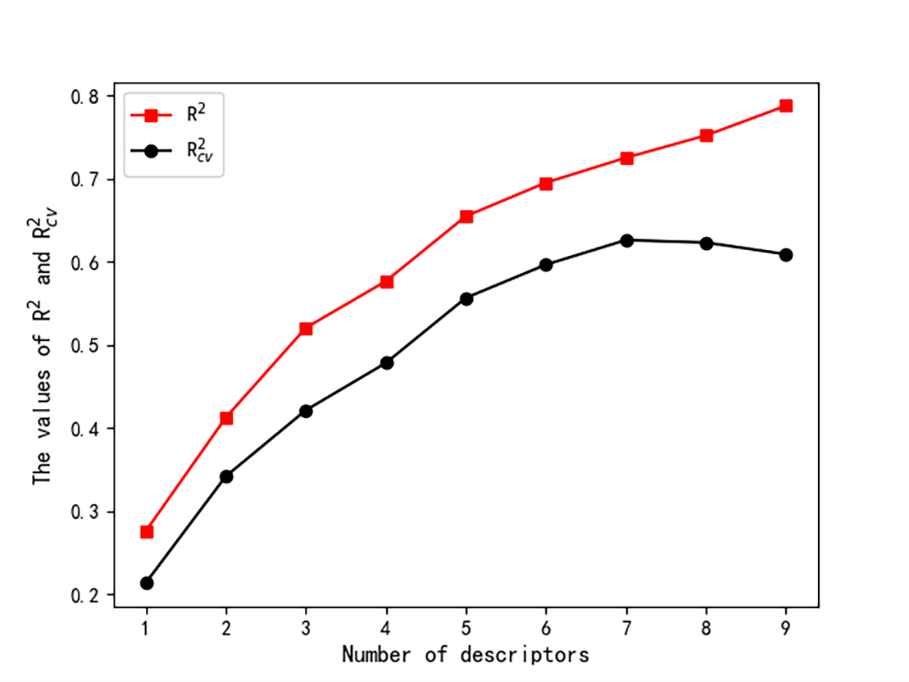


**Figure 2.** **The plot of experimental and calculated −lg ($\text{IC}_{50}$) of HM model**


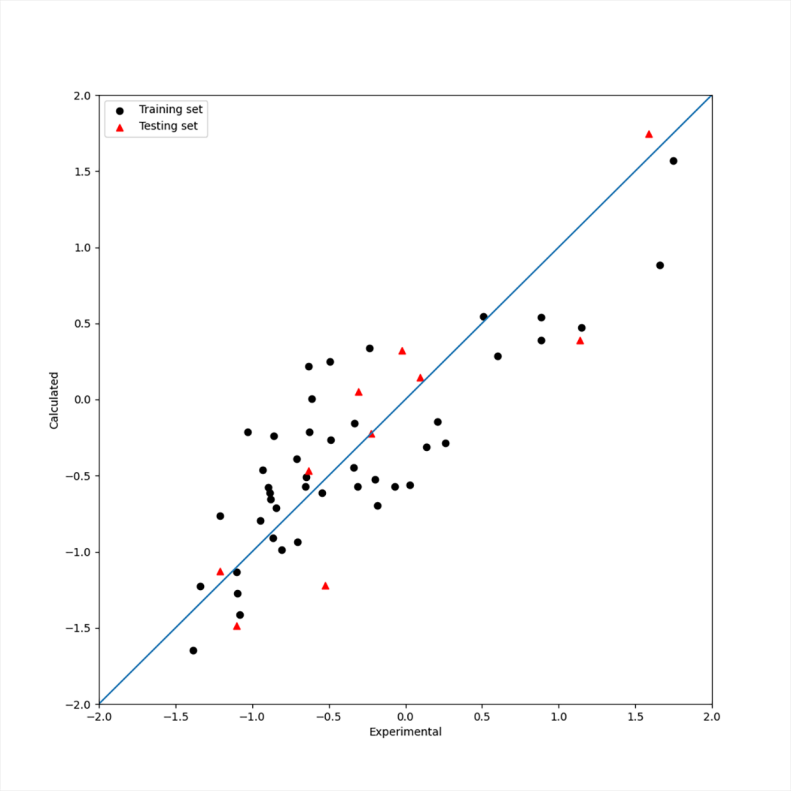


**Figure 3.** **The importance of descriptors selected by XGBoost**


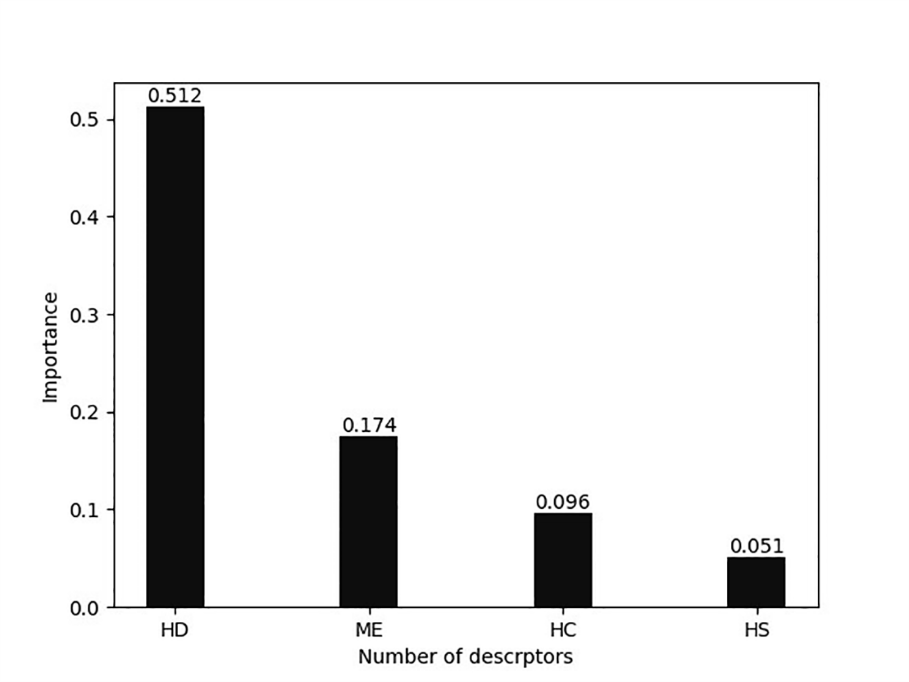


**Figure 4.** **The fitness of each iteration**


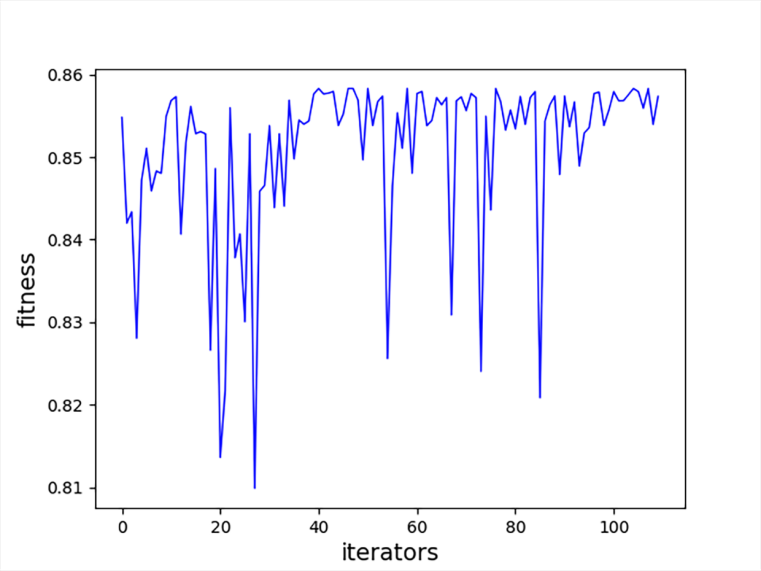


**Figure 5.** **The plot of experimental and calculated −lg ($\text{IC}_{50}$) of RF model**


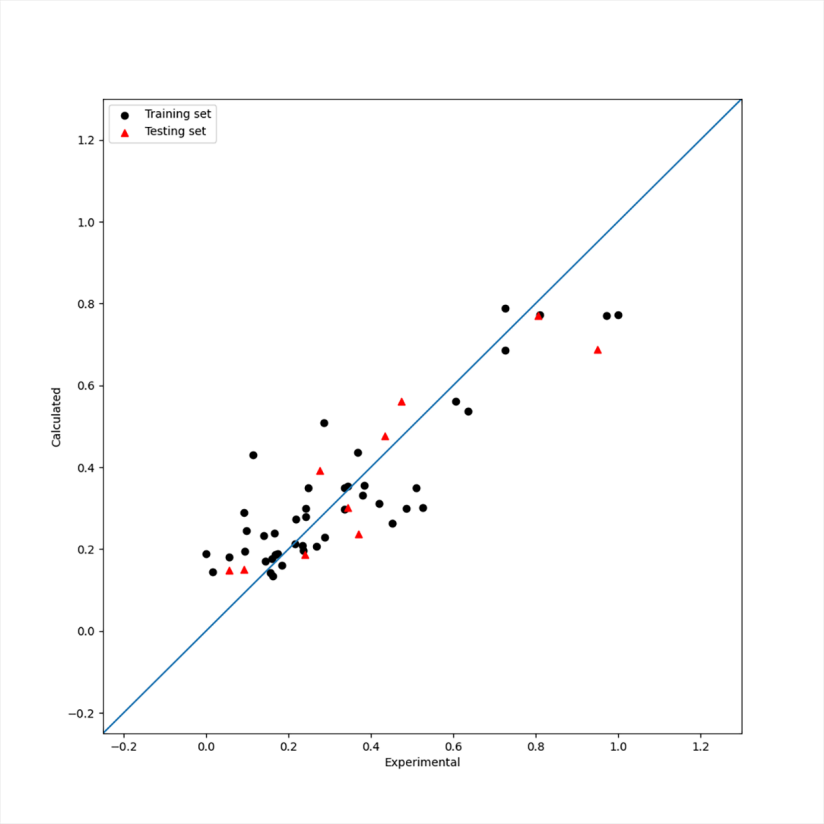


**Figure 6.** **The plot of experimental and calculated −lg ($\text{IC}_{50}$) of Linear-SVR model**


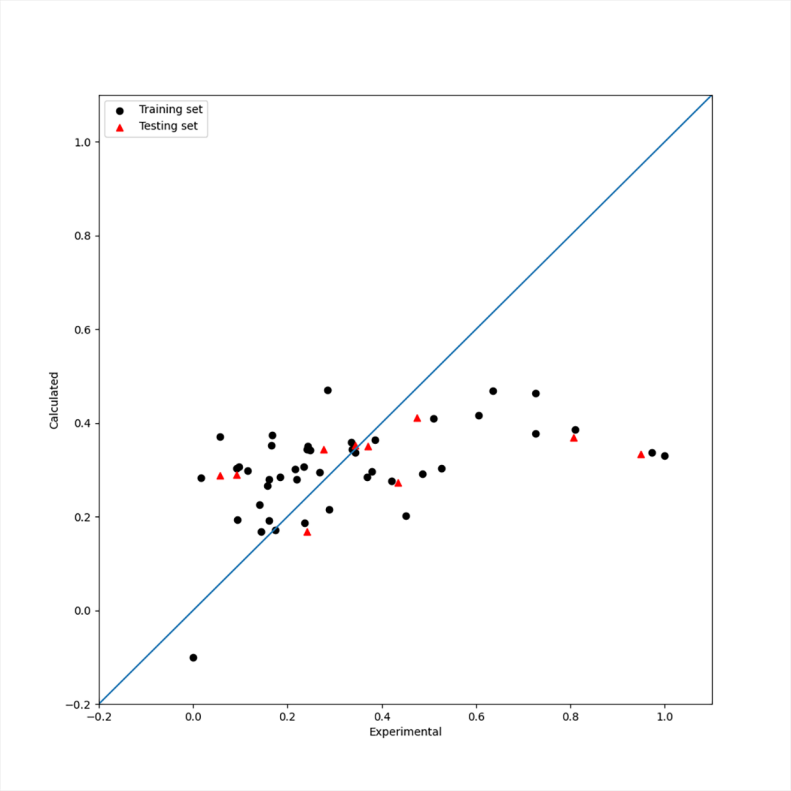


**Figure 7.** **The plot of experimental and calculated −lg ($\text{IC}_{50}$) of Poly-SVR model**


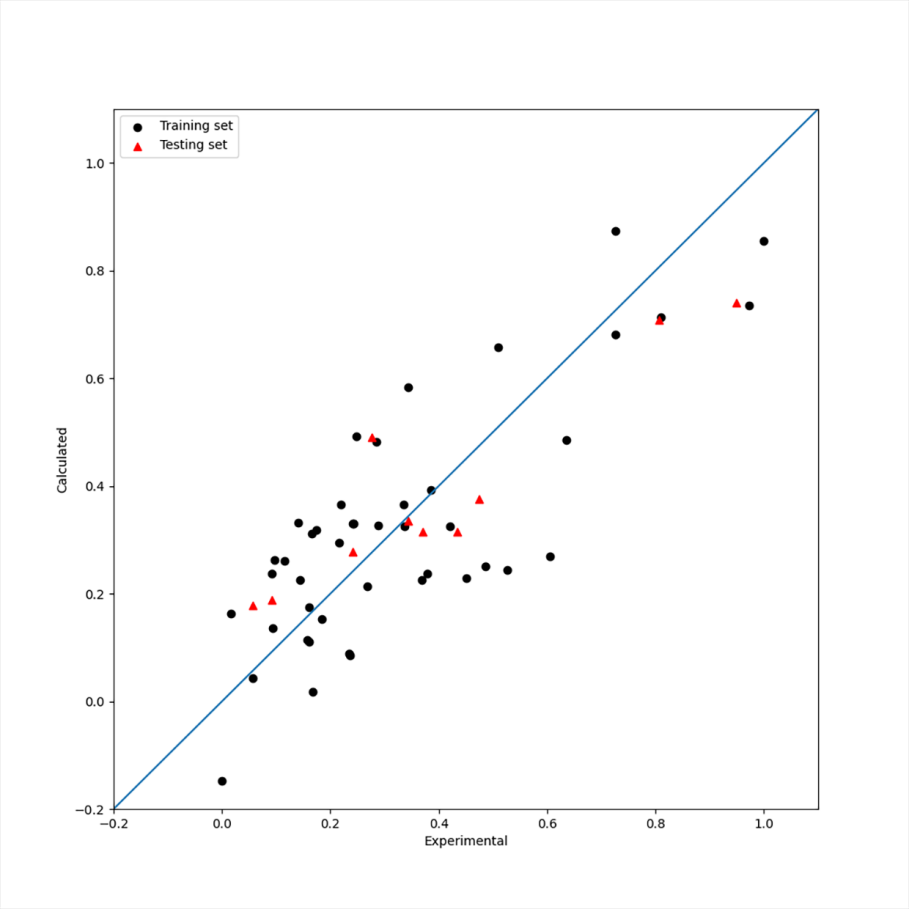


**Figure 8.** **The plot of experimental and calculated −lg ($\text{IC}_{50}$) of RBF-SVR model**


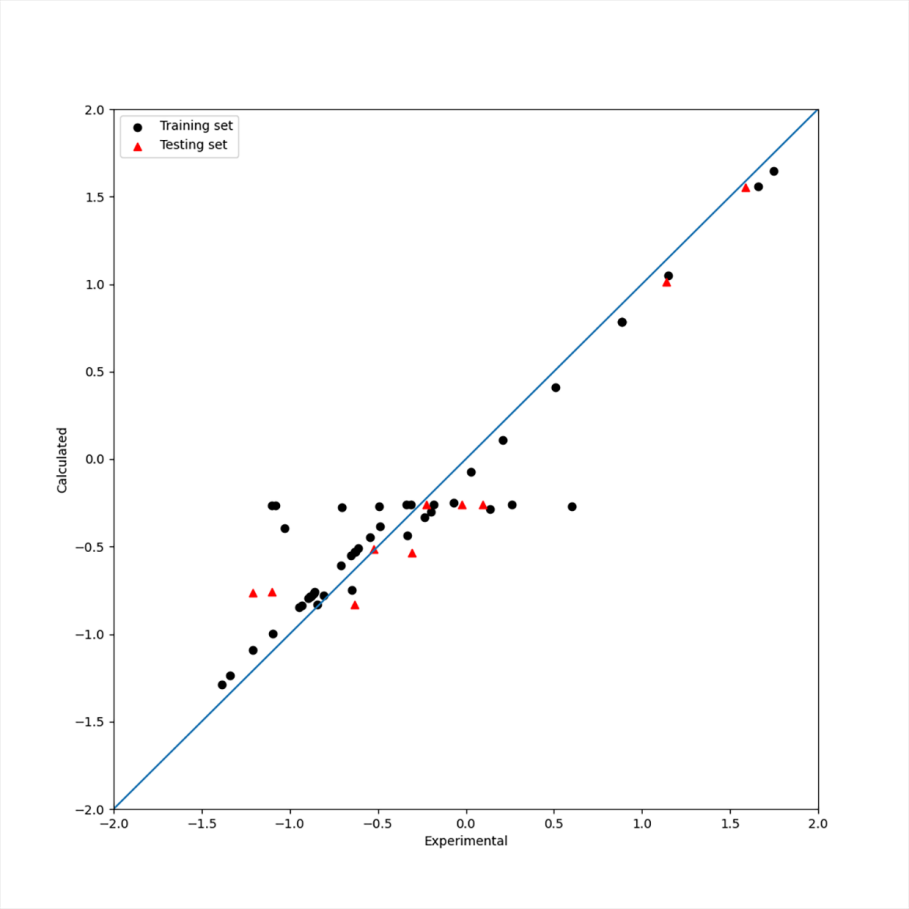


**Figure 9.** **The plot of experimental and calculated −lg (IC50) of MIX-SVR model**


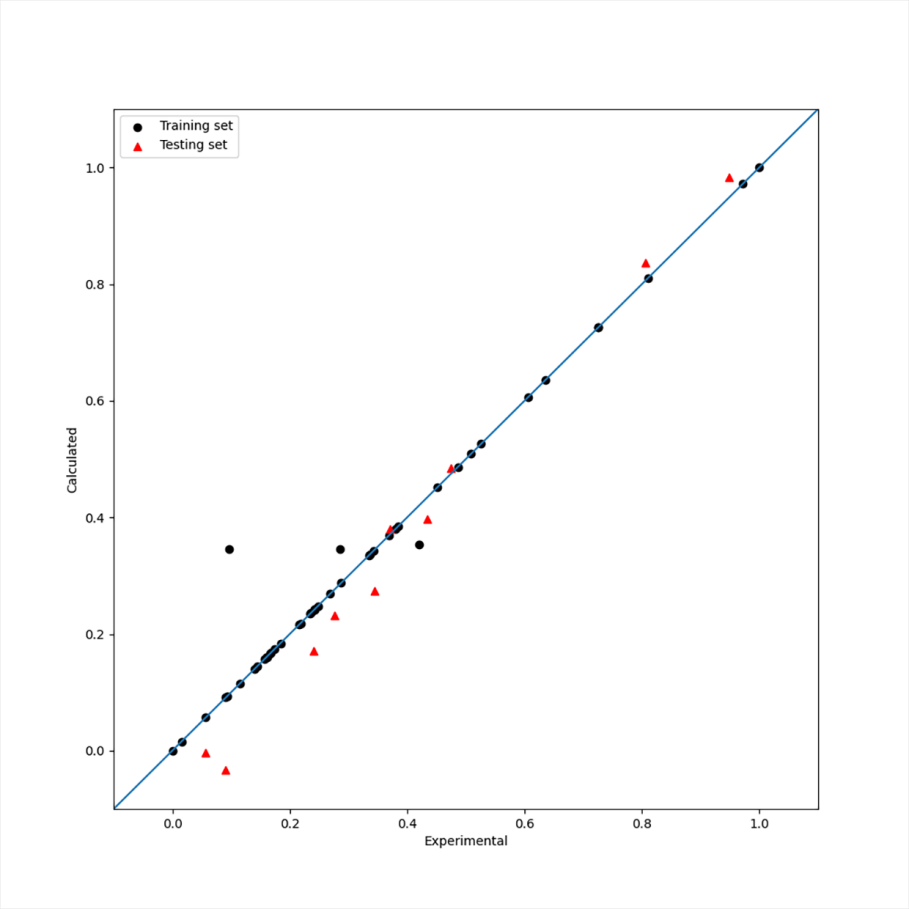

Supplement: Supplementary file 1 [file Table1.DOCX]
