# Supplementary figures and images for: Quantitative structure–activity relationship study of amide derivatives as xanthine oxidase inhibitors using machine learning
Source: Front Pharmacol. 2023 Jun 29;14:1227536. doi: 10.3389/fphar.2023.1227536 (PMC10339742; doi:10.3389/fphar.2023.1227536)

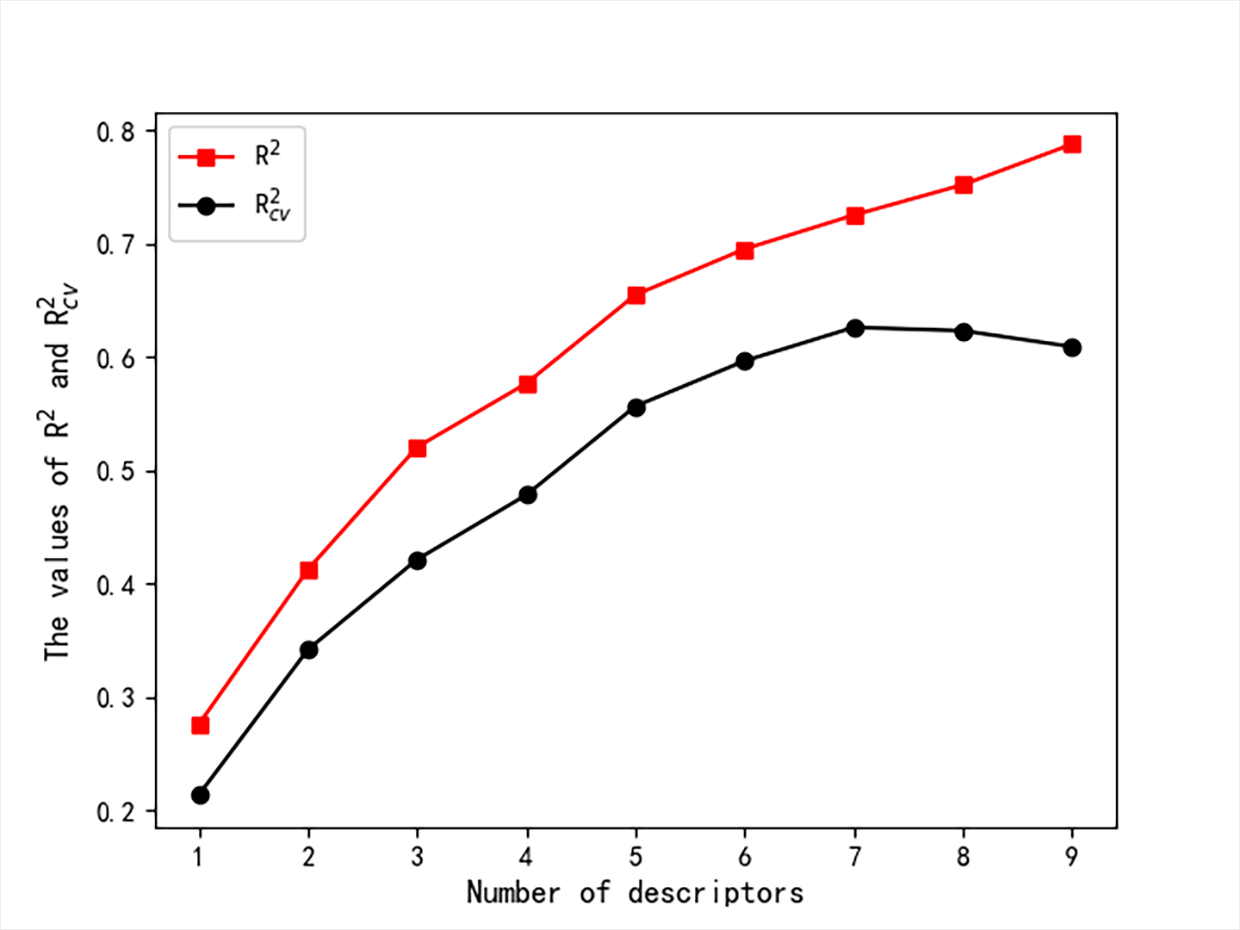

Supplement: Supplementary file 2 [file DataSheet1.ZIP › SupplementaryMaterial Presentation/Figure/Figure1.tif]

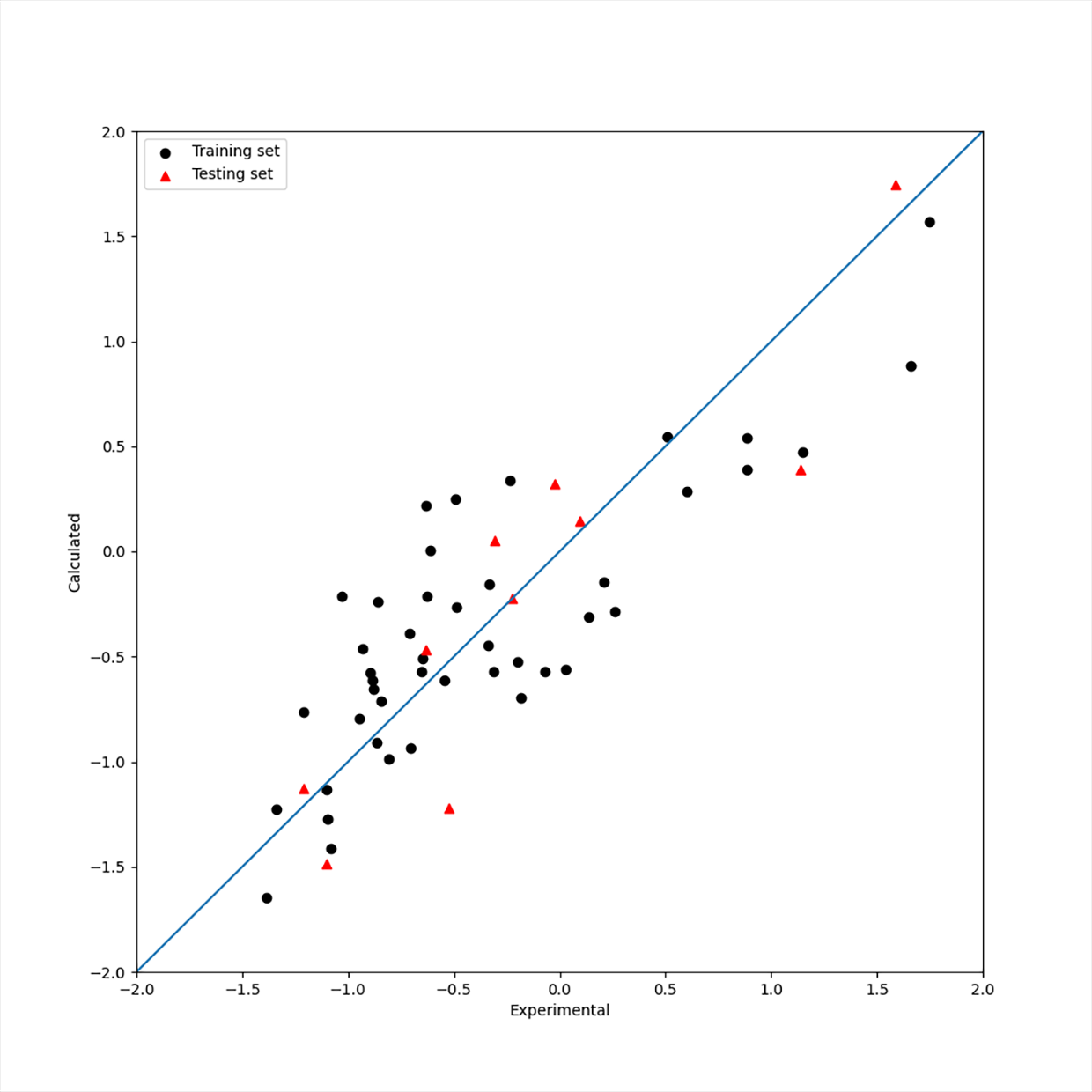

Supplement: Supplementary file 2 [file DataSheet1.ZIP › SupplementaryMaterial Presentation/Figure/Figure2.tif]

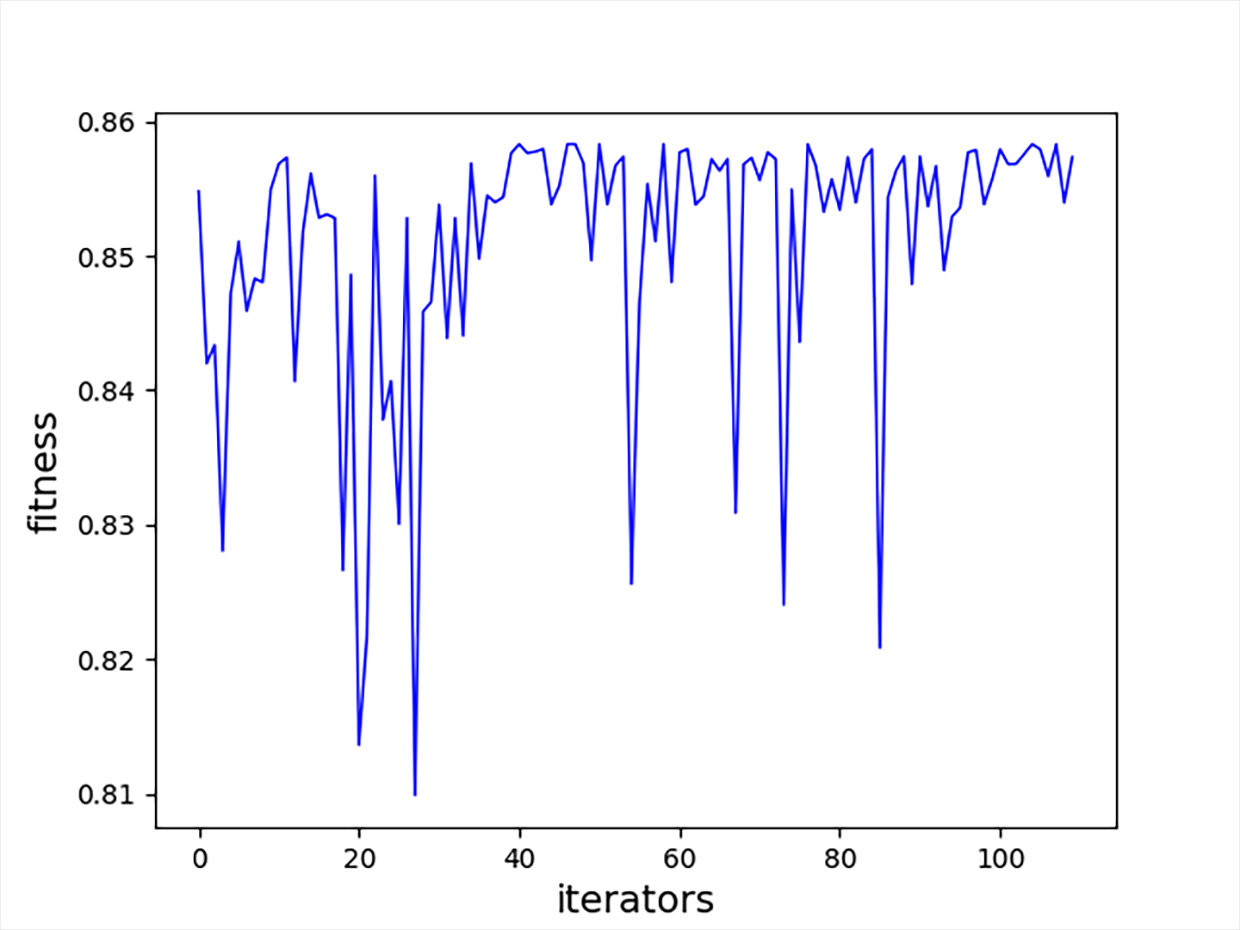

Supplement: Supplementary file 2 [file DataSheet1.ZIP › SupplementaryMaterial Presentation/Figure/Figure4.tif]

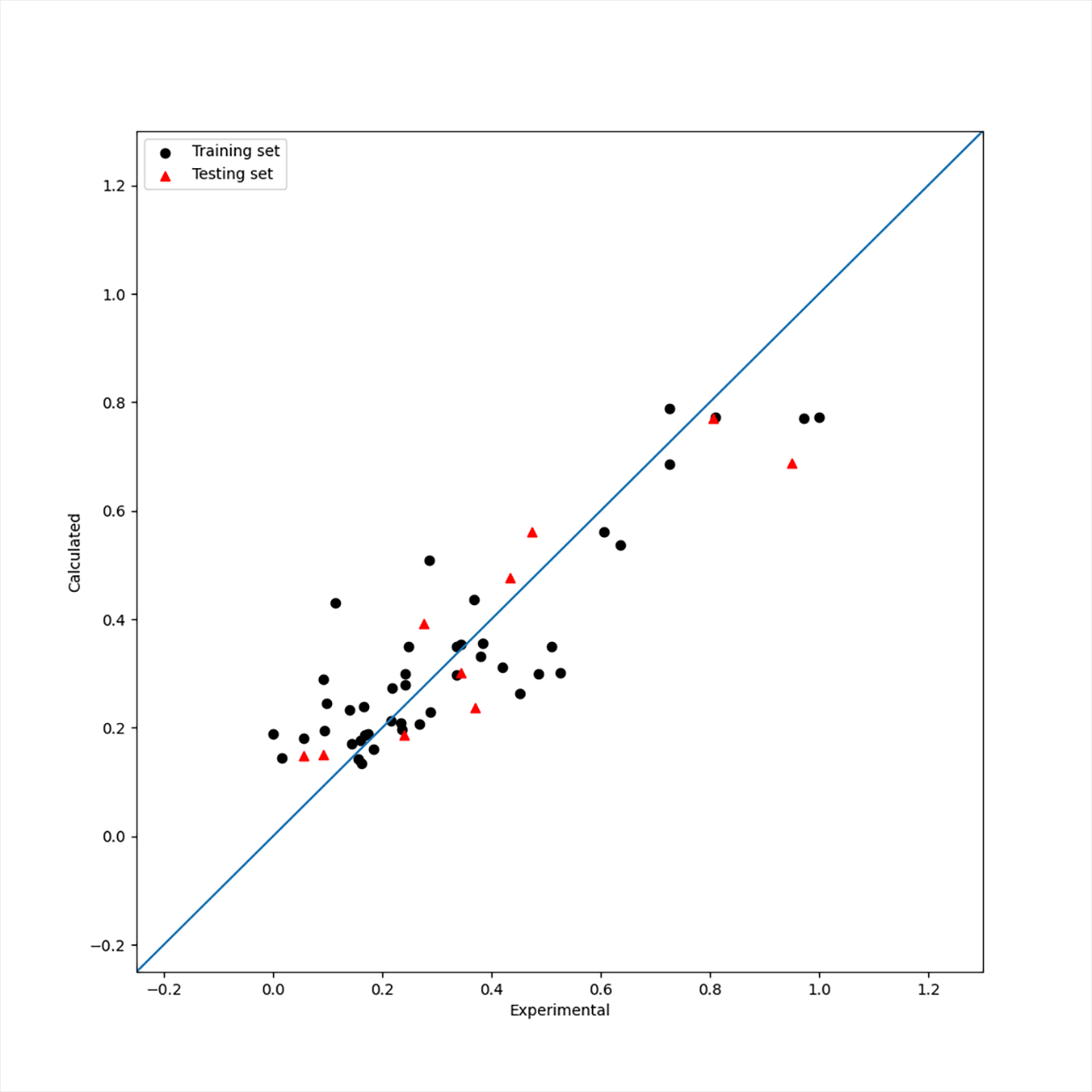

Supplement: Supplementary file 2 [file DataSheet1.ZIP › SupplementaryMaterial Presentation/Figure/Figure5.tif]

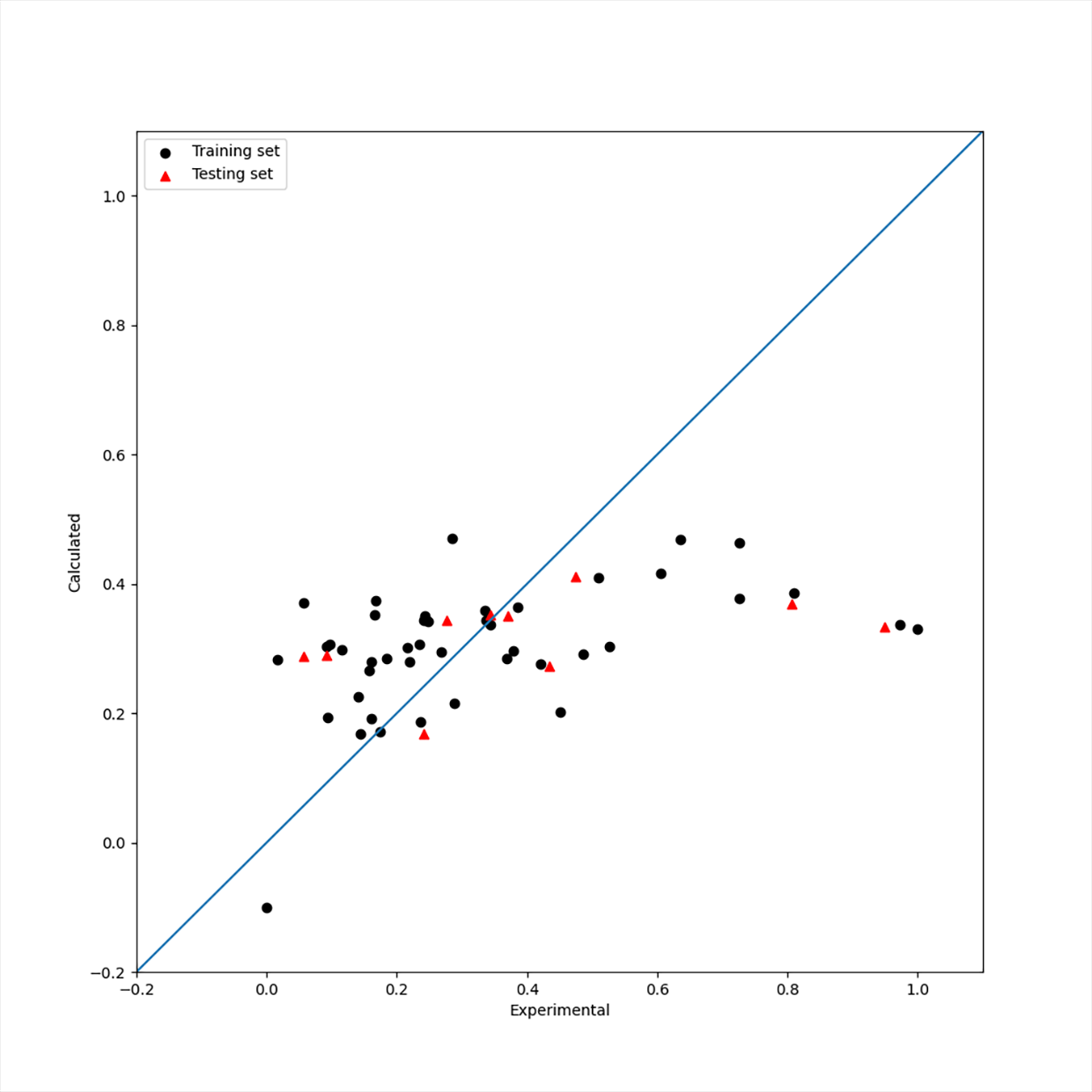

Supplement: Supplementary file 2 [file DataSheet1.ZIP › SupplementaryMaterial Presentation/Figure/Figure6.tif]

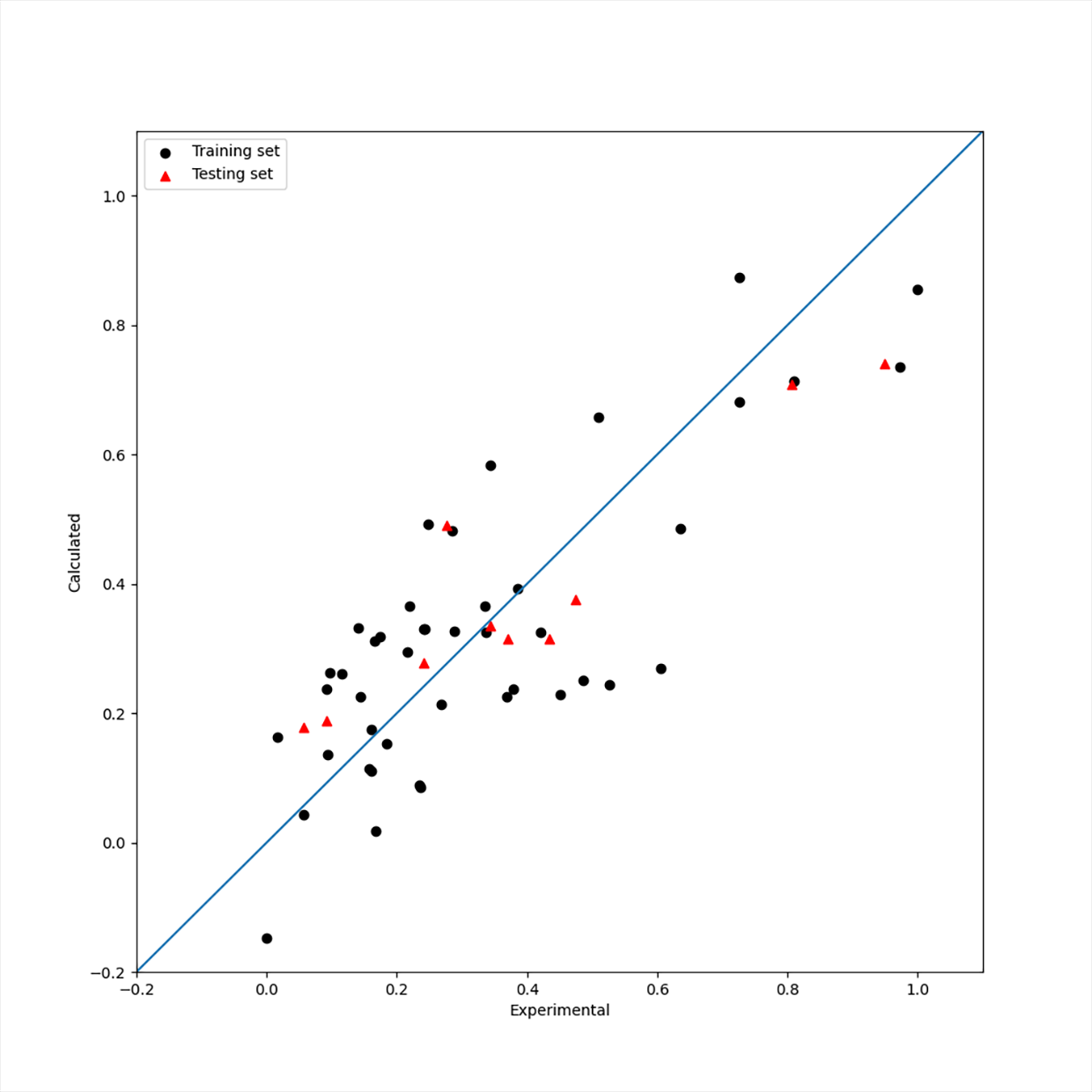

Supplement: Supplementary file 2 [file DataSheet1.ZIP › SupplementaryMaterial Presentation/Figure/Figure7.tif]

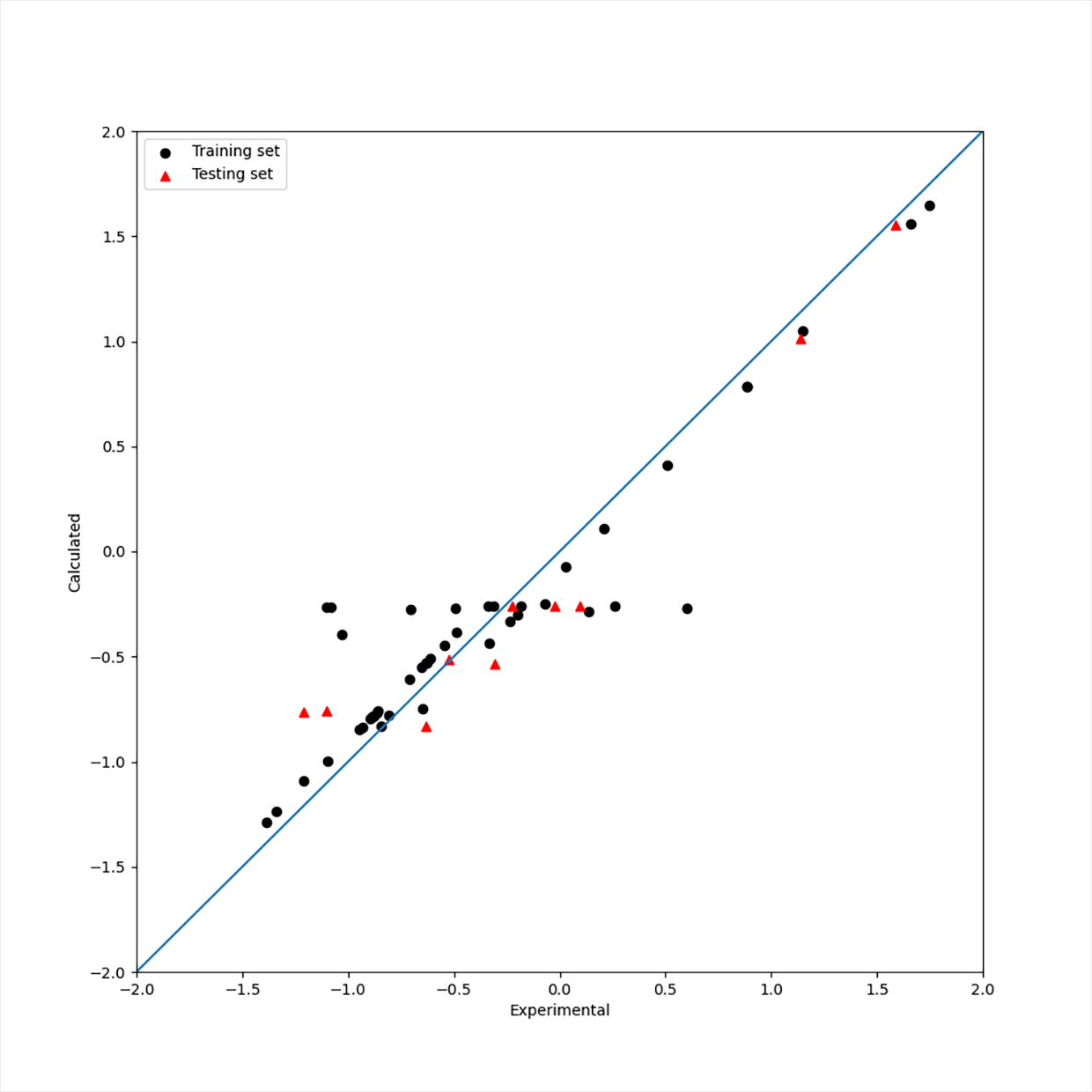

Supplement: Supplementary file 2 [file DataSheet1.ZIP › SupplementaryMaterial Presentation/Figure/Figure8.tif]

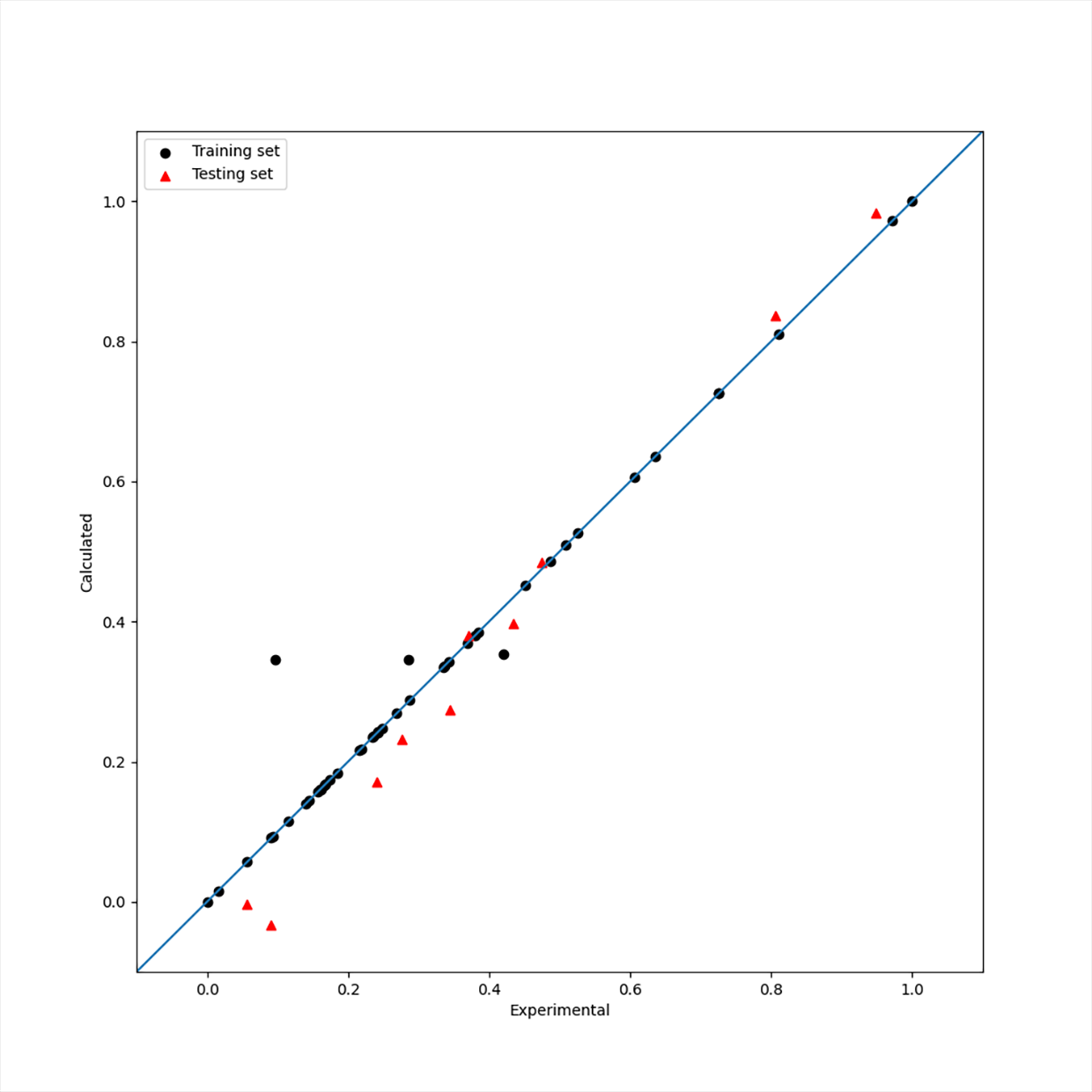

Supplement: Supplementary file 2 [file DataSheet1.ZIP › SupplementaryMaterial Presentation/Figure/Figure9.tif]
